# Hurricane Effects on a Shallow Lake Ecosystem and Its Response to a Controlled Manipulation of Water Level

**DOI:** 10.1100/tsw.2001.14

**Published:** 2001-04-04

**Authors:** Karl E. Havens, Kang-Ren Jin, Andrew J. Rodusky, Bruce Sharfstein, Mark A. Brady, Therese L. East, Nenad Iricanin, R. Thomas James, Matthew C. Harwell, Alan D. Steinman

**Affiliations:** South Florida Water Management District, 3301 Gun Club Road, West Palm Beach, Florida 3346-4680, USA

**Keywords:** shallow lakes, submerged aquatic vegetation, hurricanes, wind effects, water level control, lake recession, whole-ecosystem experiment, sediment-water interactions

## Abstract

In order to reverse the damage to aquatic plant communities caused by multiple years of high water levels in Lake Okeechobee, Florida (U.S.), the Governing Board of the South Florida Water Management District (SFWMD) authorized a "managed recession" to substantially lower the surface elevation of the lake in spring 2000. The operation was intended to achieve lower water levels for at least 8 weeks during the summer growing season, and was predicted to result in a large-scale recovery of submerged vascular plants. We treated this operation as a whole ecosystem experiment, and assessed ecological responses using data from an existing network of water quality and submerged plant monitoring sites. As a result of large-scale discharges of water from the lake, coupled with losses to evaporation and to water supply deliveries to agriculture and other regional users, the lake surface elevation receded by approximately 1 m between April and June. Water depths in shoreline areas that historically supported submerged plant communities declined from near 1.5 m to below 0.5 m. Low water levels persisted for the entire summer. Despite shallow depths, the initial response (in June 2000) of submerged plants was very limited and water remained highly turbid (due at first to abiotic seston and later to phytoplankton blooms). Turbidity decreased in July and the biomass of plants increased. However, submerged plant biomass did not exceed levels observed during summer 1999 (when water depths were greater) until August. Furthermore, a vascular plant-dominated assemblage (Vallisnera, Potamogeton, and Hydrilla) that occurred in 1999 was replaced with a community of nearly 98% Chara spp. (a macro-alga) in 2000. Hence, the lake’s submerged plant community appeared to revert to an earlier successional stage despite what appeared to be better conditions for growth. To explain this unexpected response, we evaluated the impacts that Hurricane Irene may have had on the lake in the previous autumn. In mid-October 1999, this category 1 hurricane passed just to the south of the lake, with wind velocities over the lake surface reaching 90 km h-1 at their peak. Output from a three-dimensional hydrodynamic / sediment transport model indicates that during the storm, current velocities in surface waters of the lake increased from near 5 cm s to as high as 100 cm s. These strong velocities were associated with large-scale uplifting and horizontal transport of fine-grained sediments from the lake bottom. Water quality data collected after the storm confirmed that the hurricane resulted in lake-wide nutrient and suspended solids concentrations far in excess of those previously documented for a 10-year data set. These conditions persisted through the winter months and may have negatively impacted plants that remained in the lake at the end of the 1999 growing season. The results demonstrate that in shallow lakes, unpredictable external forces, such as hurricanes, can play a major role in ecosystem dynamics. In regions where these events are common (e.g., the tropics and subtropics), consideration should be given to how they might affect long-term lake management programs.

## INTRODUCTION

The dynamics of shallow lakes are complex and generally less predictable than in deeper stratified ecosystems. These reflect the close coupling of physical, chemical, and biological processes in the water column and sediments of shallow lakes, lack of a stable period of summer stratification, and the sometimes-dramatic effects of seasonal and stochastic events such as windstorms. In these lakes, sediment-water interactions can play the major role in determining water column nutrient concentrations[1], irradiance profiles[2], and ability of the system to support different types of primary producers[3]. Major interactions between the benthos and water column include physical resuspension of sediments by wind[2]; sorption and desorption of nutrient elements from resuspended particles[4]; diffusion of nutrients into anoxic water columns[1]; internal nutrient loading due to bioturbation[5]; and uptake and release of nutrients by vascular plants and periphyton[6–8].

The timing and magnitude of sediment-water interactions in shallow lakes are governed in part by three physical conditions — water depth, wind velocity, and fetch. Depending on the prevailing conditions, shallow eutrophic lakes influenced by these factors could display two dramatically different states (clear vs. turbid), as predicted by mathematical models[9] and documented by field observations[10,11]. Clear lakes generally have high densities of submerged aquatic plants and low concentrations of seston, while the opposite tendencies occur in turbid lakes. A major tenet of the alternative state model[9] is that shallow lakes with high nutrient loading can "switch" their state if impacted by a strong external or internal force. Factors commonly mentioned include a dramatic change in water level[10], a major flushing event[12], or a dramatic reduction in benthivorous fish stock[13]. In regions that are influenced by hurricanes, strong wind events could represent another force that could switch a lake from a clear to turbid state. For several reasons, including unpredictable timing and location, risk of damage to field equipment and observers, hurricane impacts on shallow lakes are not well documented. There has been speculation that a hurricane caused at least one large nutrient-enriched lake (Lake Apopka, Florida) to switch from clear and macrophyte dominated to turbid and dominated by phytoplankton[14,15]. The triggering event was said to have occurred in the early 1940s. Similarly, a decline of macrophytes in Chesapeake Bay, U.S. was related to the possible effects of a tropical storm[16].

We have been studying ecosystem dynamics in a large shallow lake in south Florida, U.S. (Lake Okeechobee) and previously described how this lake changed as a function of cultural eutrophication[17]. During autumn 1999, while our intensive water quality and vegetation monitoring programs were in place, the lake was impacted by a hurricane (Irene). The storm had dramatic effects on the ecosystem, influencing how the lake responded to a managed lake recession operation carried out in April-May 2000. Here we report on these responses, and offer insight into how windstorms might affect the dynamics of large shallow lakes and their responses to management actions.

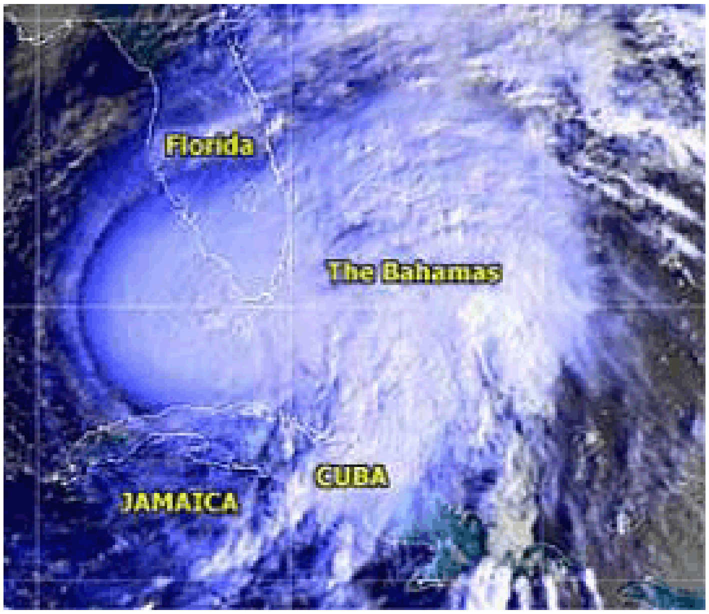

Hurricane Irene

## STUDY SITE

Lake Okeechobee is one of the world's largest subtropical lakes, with a surface area of approximately 1,800 km$^2$. The lake is located in the peninsula of Florida, U.S., where it occurs at the center of both the regional aquatic ecosystem and a large flood control project constructed in the mid-1900s by the U.S. Army Corps of Engineers. Despite its large size, the lake is very shallow (mean depth ~3 m), and water depths vary considerably as a function of rainfall, flood control discharges, and water supply deliveries. The lake is considered eutrophic based on its high concentrations of nutrients and phytoplankton, and it is turbid due to frequent wind resuspension of mud sediments that predominate near the lake's center[17]. Along the western shoreline (Fig. 1) there is a 400-km$^2$ littoral marsh with a mixed community of submerged and emergent aquatic vegetation. Immediately adjacent to the marsh is a 200-km$^2$ near-shore region (0.5 to 2 m deep) where submerged plants can sometimes become abundant. Beyond that region is a 1,200-km$^2$ pelagic zone (3 to 5 m deep) that supports only planktonic producers.

The watershed surrounding Lake Okeechobee (22,500 km$^2$) is basically a shallow trough that drains south from Orlando to the Florida Everglades, and is bounded by the sand hills of the Lake Wales Ridge on the west and the

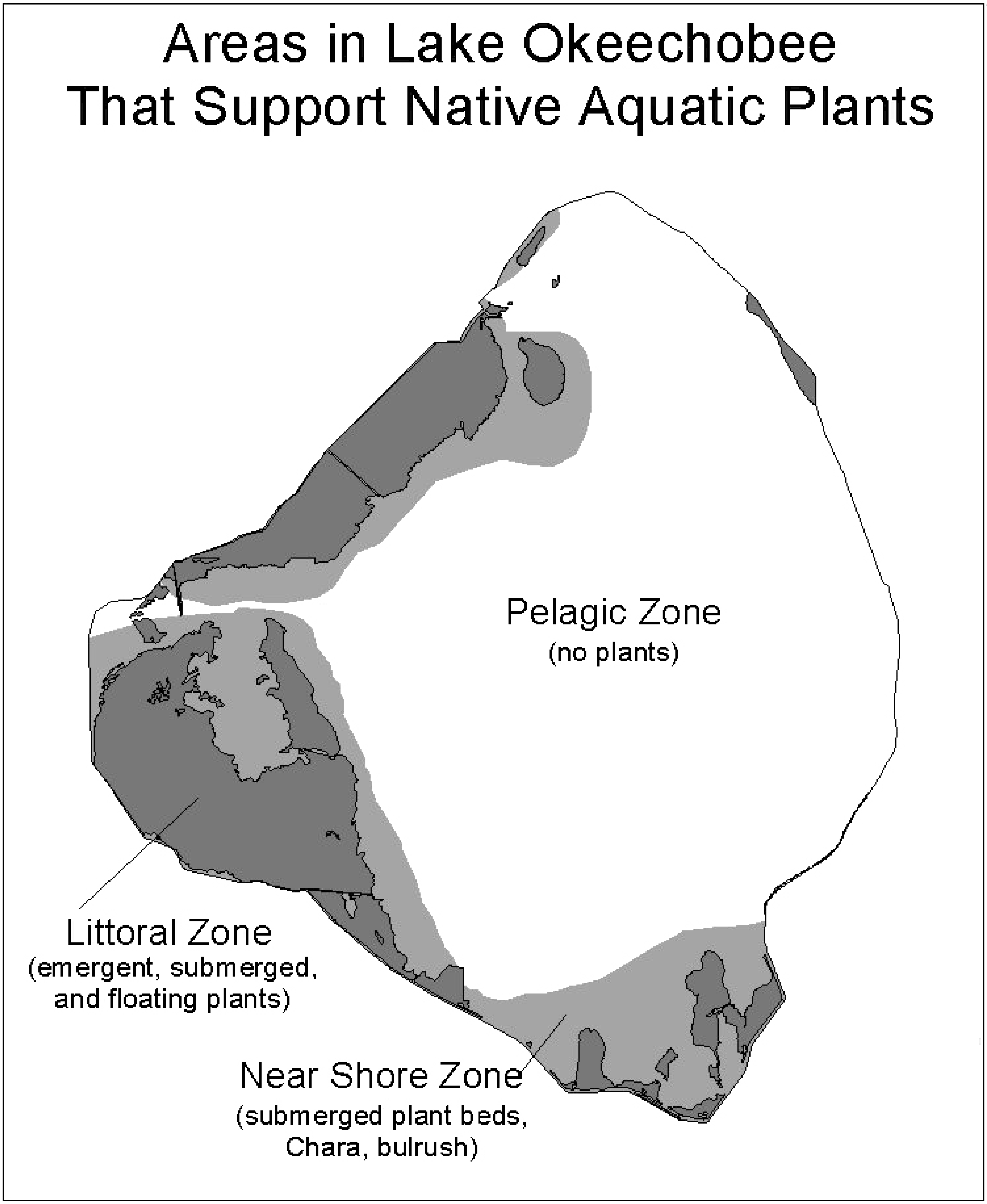

**FIGURE 1**. Map of Lake Okeechobee, Florida, U.S., showing regions that support distinct communities of primary producers, largely due to variations in water depth, which increases from below 1 m in the littoral zone to near 5 m in the pelagic zone.

upland marshes of the Osceola Plain on the east. The hydrology is characterized by low-gradient, poorly drained landscapes with many marshes and sloughs. The watershed receives 120 cm of rainfall per year (with considerable year-to-year variation); most rainfall occurs in summer, between May and October. The watershed has been heavily developed for agricultural purposes, including beef cattle ranching, dairy farming, citrus, and other agricultural crops. Small urban areas occur on the north, west, and south sides of the lake, but they are not directly in contact with the shoreline due to a large man-made dike that surrounds the lake. All inflows to the lake (except one) and all outflows pass through gates, pumps, or other structures.

## METHODS

In this report we synthesize information from a variety of ecosystem sampling programs from before and during the lake recession period, as well as output from geographic information systems (GIS) and a lake hydrodynamic / sediment transport model. The focus is on ecosystem changes that occurred coincident with the recession, and in particular, on how unexpected responses were likely caused by the impacts of a hurricane whose eye passed just to the south of the lake in October 1999.

Regular water quality sampling is carried out on the lake by the South Florida Water Management District (SFWMD), on a monthly basis from October to April and twice monthly from May to September. Approximately 30 sites are sampled in the pelagic and near-shore areas, and surface water samples are analyzed for chemical constituents according to standard U.S. Environmental Protection Agency methods[18,19]. Transparencies are measured using a 20-cm black and white Secchi disk. Based on the results from each sampling event, isoline maps are created with Surfer[Ò], in order to identify the spatial distributions of major attributes. The data used for this paper include total phosphorus (TP) concentrations and Secchi transparencies during October-November 1999 (the hurricane period) and April-May 2000 (the lake recession period).

Submerged aquatic vegetation (SAV) sampling on the lake occurred for a brief period in the late 1980s[20], after which those data were not collected until 1999. In April 1999 we began a regular program to survey this community. Of the 60 near-shore transects that were surveyed in the earlier program, we selected 14 that encompassed the near-shore region. Along each transect, 3 equidistant sites were identified and regularly sampled (42 sites total). The length of each transect was determined so that it extended from the lakeshore outward to the farthest location where submerged plants were found in the 1989 project. Sampling was done in April, July, and October 1999, and in January, April-August, and October 2000. The increased frequency of sampling in summer 2000 was driven by a desire to closely monitor responses to the lake recession operation. After navigating to each site using a global positioning system (GPS), plant material was collected by destructive harvesting. First a diver entered the water and visually surveyed a large area of the lakebed to determine if plants were present. If a site contained vascular plants, three 0.5-m$^2$ plastic grids were randomly thrown from the boat and divers manually removed all aboveground biomass contained within. At sites where the benthic macro-alga *Chara* spp. occurred, three additional samples were collected using a Ponar dredge to characterize the biomass of this low-growing species. In the laboratory all plant material was sorted to species (except *Chara* spp.), dried in an oven at 60$^o$C, and then weighed to determine dry mass (g m$^{-2}$).

At the end of the 2000 growing season a more intensive survey was conducted to determine the spatial extent of various submerged plants. The entire lake surface was subdivided into a 500- ´ 500-m regular grid and the coordinates of a central sampling point in each grid cell were identified in GIS. In September-October, sites in the near-shore zone were navigated to by GPS and the presence of plants determined by taking three samples with a hinged pair of garden rakes that sampled approximately 0.33 m$^2$ (total sample ~1 m$^2$) of the lake bottom. Sampling occurred from shoreline towards deeper water until at least two successive cells were encountered with no plants. This approach defined the deep-water limits of the sampling effort and ultimately included 1,684 near-shore cells.

In order to identify potential effects of the October 1999 hurricane on lake currents, sediment transport, and water quality, a three-dimensional hydrodynamic and sediment transport model[21] based on the Environmental Fluid Dynamics Code[22] was used. This model simulates water currents in the lake over a 22,928 unit grid of 1- ´ 1-km cells, and when coupled with a wind-wave model, can provide output including suspended sediment concentrations. Model output is used here to illustrate differences in surface currents and resuspended sediment concentrations that occurred just before, during, and after the hurricane event. Input for the hydrodynamic model included wind velocities, air temperatures, and incident solar radiation, measured at permanent monitoring structures in the lake maintained by the SFWMD. These data are available online at www.sfwmd.gov/curre/2_techdata.html.

## RESULTS AND DISCUSSION

### Recent History of High Water and Its Ecological Effects

During the 28 years that the SFWMD has been collecting water quality data on Lake Okeechobee, lake stage has varied considerably (Fig. 2), including two drought periods when lake stage dropped to near 3 m, and periods of high rainfall and surface water inputs when stage exceeded 5.5 m. In the late 1980s, after two successive years of low lake stage, nearly 14,000 ha of submerged vegetation was identified[23] by remote sensing in the near-shore region (Fig. 3a). Water clarity during this period also was high. In recent years the near-shore region has been largely devoid of plants (Fig. 3b), and has been characterized by deeper (by up to 1 m), more turbid water[8,24]. Previous studies have shown that the biomass of submerged plants in this lake is negatively correlated with water depth and positively correlated with water transparency[24,25]. These results are consistent with findings from shallow lake research in the Netherlands[12].

When submerged plants decline because of deep and/or turbid water, a positive feedback loop may ensue that maintains turbid conditions. Without plants to stabilize sediments, there can be increased sediment resuspension by wind and waves and no competition for nutrients by phytoplankton[26]. These conditions prevent plant recovery, and the underlying cycle is difficult to break[9]. Only strong actions, either natural or induced by humans, can make conditions favorable again for plant growth and development of an alternative stable clear-water state.

In addition to loss of submerged plants, the emergent plant community was damaged by high water in Lake Okeechobee[27], because wind-driven waves eroded up to 300 m of emergent plant beds along the western shoreline. Large "berms" of detritus and dead plant material became prominent along the lakeshore (Fig. 4a,b) after autumn 1998 and persist today where the emergent marsh habitat once was dominated by cattail (*Typha*), bulrush (*Scirpus*), and spikerush (*Eleocharis*). Along with the submerged plants, this community provides important spawning and foraging habitat for fish[28-32].

Loss of the submerged and emergent macrophyte communities coincident with high water level led to outcries from fishermen and state and local resource managers, prompting the SFWMD to carry out the operation to lower water levels in spring 2000. In this managed recession, large volumes of water were discharged from the lake via canals to the east and west coast of Florida. At the same time there were high rates of evapotranspiration and normal seasonal use of water from the lake by agricultural and urban areas. This resulted in a decline in lake surface elevation by nearly 1 m from May to June 2000 (Fig. 2b). Lake stage then remained near 3.6 m for several months. Under these conditions, water in much of the lake's near-shore region was less than 0.5 m deep.

## Submerged Vegetation Responses

After reaching a peak occurrence at 10 of the 42 sample sites (i.e., 24% of sites) in July 1999, submerged vegetation was found at just 8, 6, and 2 locations in October 1999 and January and April 2000, respectively (Fig. 5a). We predicted a rapid response of the community to the lake recession operation, but in June and July the number of sites with vegetation was similar to that found in 1999, when water levels were higher. A dramatic increase in the spatial extent of submerged plants did not occur until August 2000. In terms of spatial distribution, just 2 western locations had sparse plants in April and this expanded to 23 sites around the lakeshore

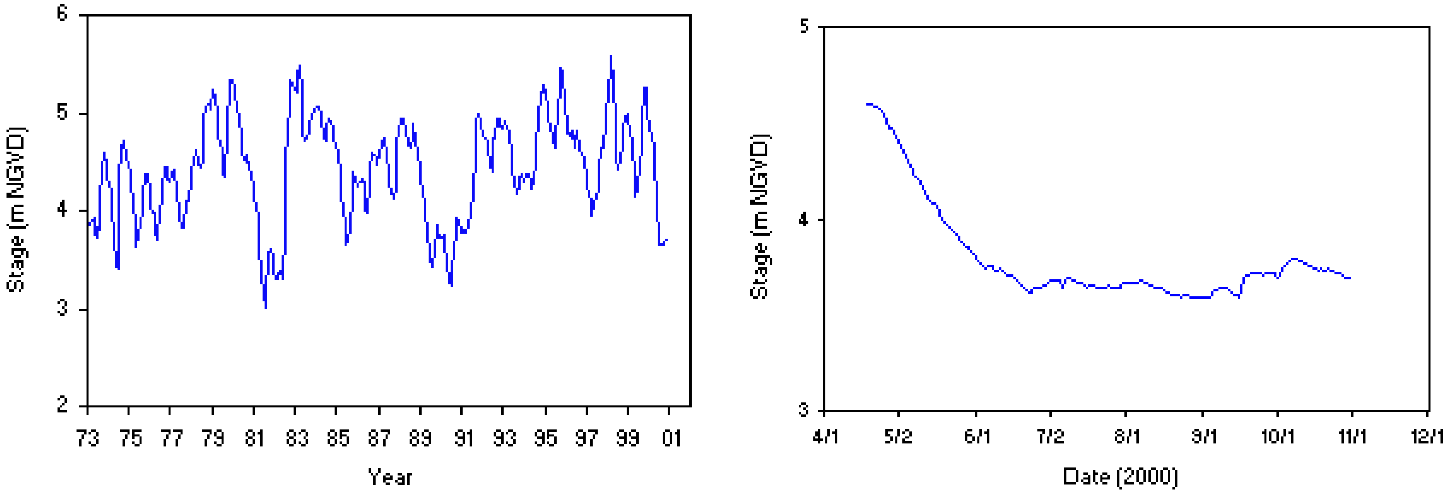

(2a) (2b)

**FIGURE 2.** Monthly lake stage in Lake Okeechobee during the period 1973 to 2000 (a) and daily lake stage during the lake recession period from May to November 2000 (b).

with sparse or dense plants in August (Fig. 6a-b). In October, a total of 22 sites continued to support plants. In the case of July-October 2000, the percentage of sites with plants actually was higher than these numbers suggest, because 9 of the 42 sites could not be sampled due to extreme low water (some were completely dry).

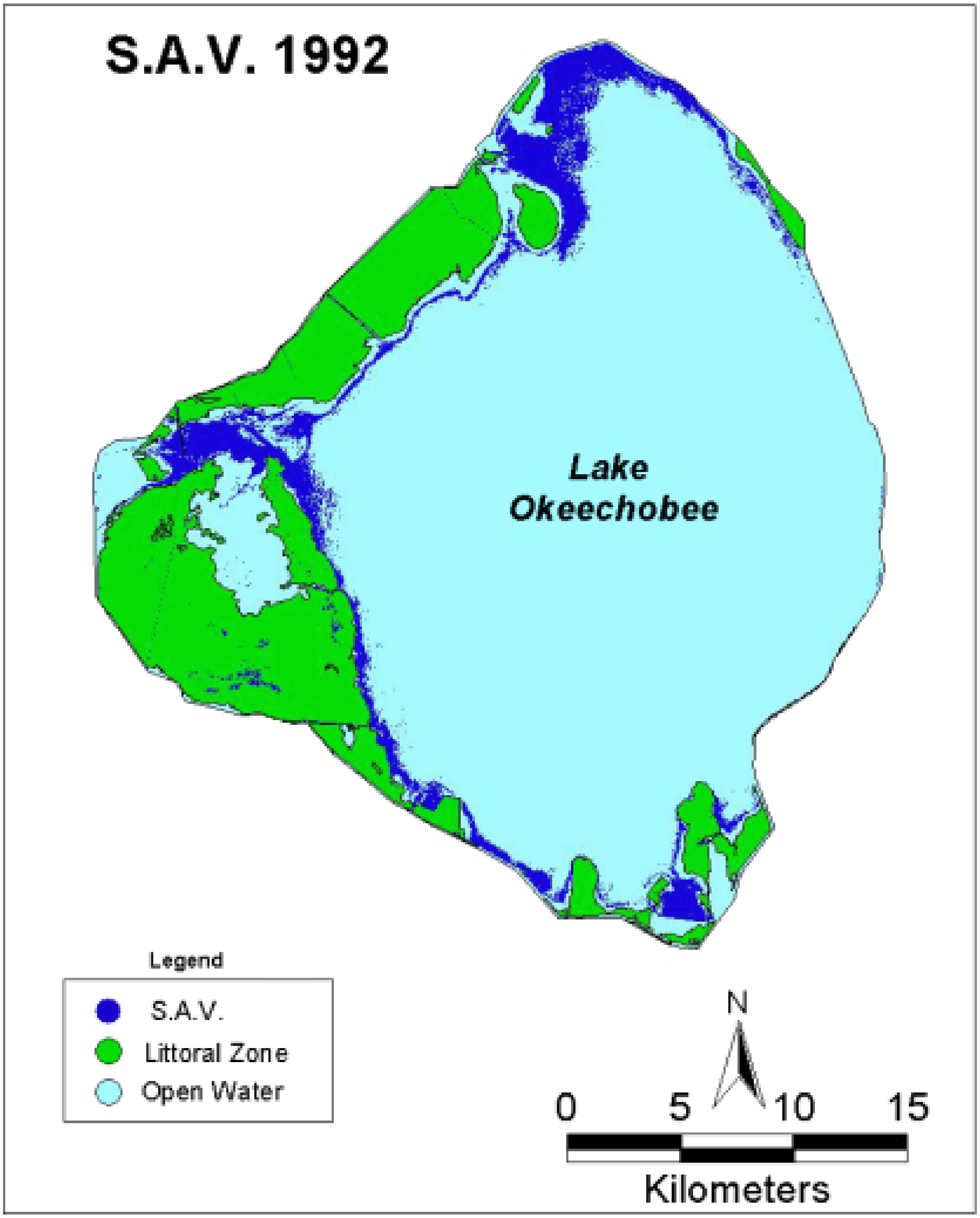

(3a)

**FIGURE 3**. Spatial distribution of submerged aquatic vegetation (blue) determined by remote sensing in summer 1989 (a), and by sampling at 42 fixed locations in summer 1999 (b).

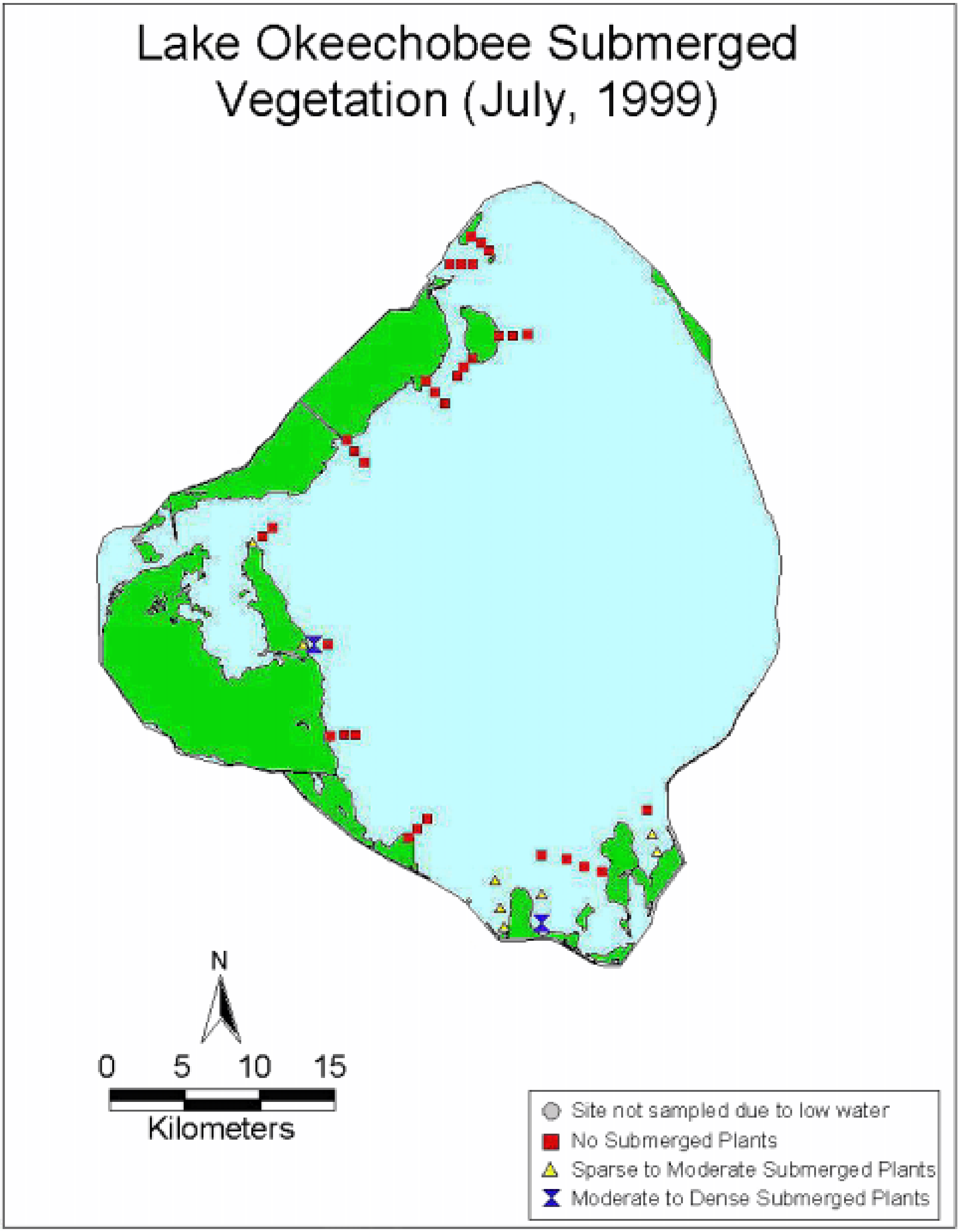

Lake Okeechobee Submerged Vegetation (July, 1999)
N
0 5 10 15
Kilometers
Site not sampled due to low water
No Submerged Plants
Sparse to Moderate Submerged Plants
Moderate to Dense Submerged Plants

**(FIGURE 3b)**

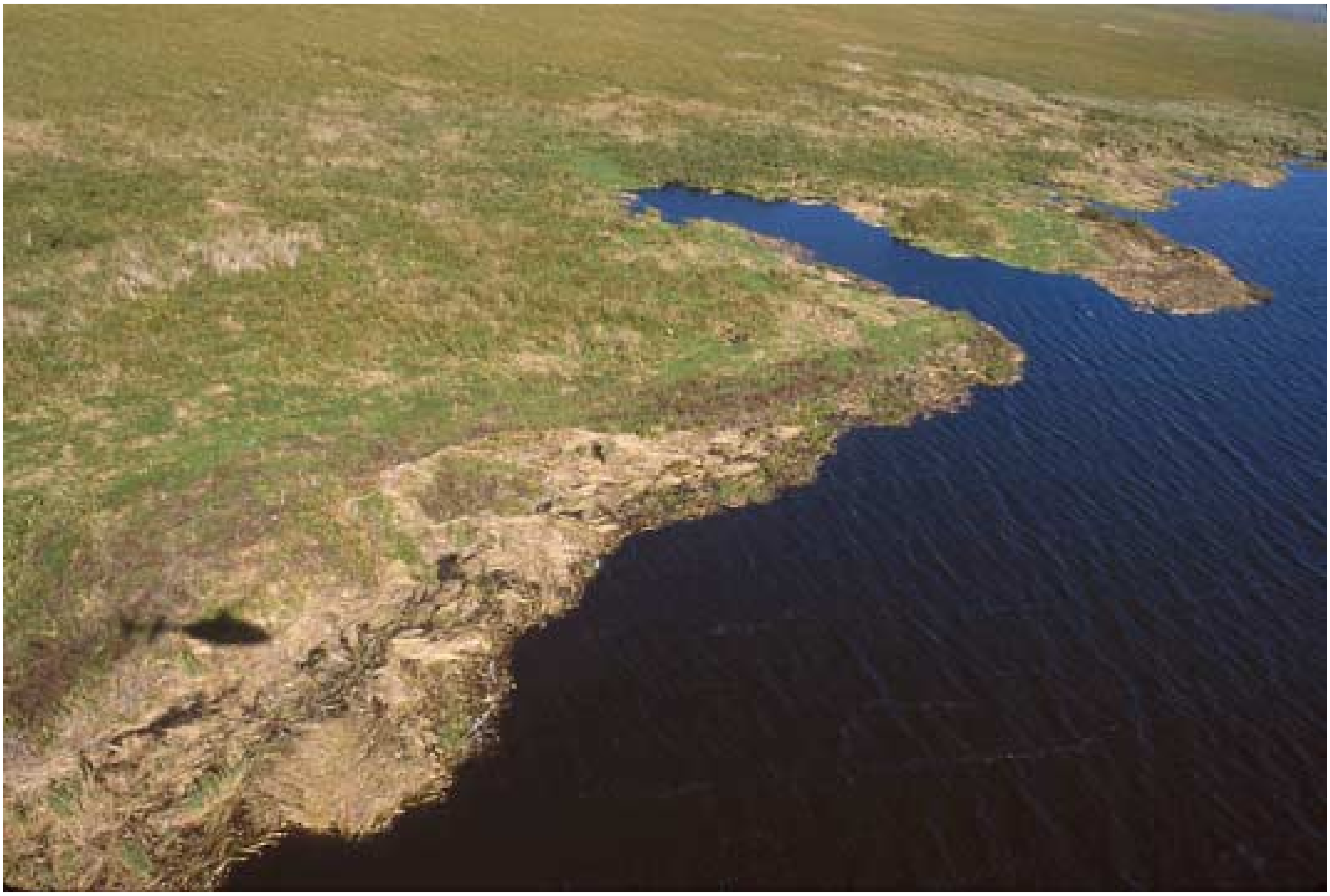

**(4a)**

**FIGURE 4**. Aerial photograph of a dense berm of sediment and dead plant material that developed along the western shoreline in autumn 1998 (a), and close-up photograph of the berm material (b).

One major factor that may have contributed to this delayed response is the high turbidity in the near-shore region that persisted from mid-October 1999 (just after the hurricane) until 1 month after the recession event in June 2000. Initially inorganic solids, as evidenced by high concentrations of total suspended solids (TSS) and nonvolatile solids (NVSS) in the water dominated the turbidity. When solids concentrations declined, there were widespread blooms of phytoplankton in the near-shore area. These were dominated by *Anabaena* spp., nitrogen-fixing cyanobacteria that have been responsible for past bloom events in this and other eutrophic lakes[33,34]. High concentrations of soluble nutrients that remained in the water column in spring 2000, coupled with the increase in underwater irradiance that occurred when solids concentrations declined, created an advantageous environment for these algae. At that time there also were few primary producers (benthic algae or plants) to compete with the phytoplankton for nutrients. Initially there was some concern among scientists and managers that the near-shore region could have developed a stable phytoplankton-dominated state. However, as the growing season progressed, benthic lawns of the macro-alga *Chara* and the filamentous alga *Lyngbya* began to develop and expand outward (see below) and the algal blooms subsided. The dense benthic communities could have reduced nutrient availability in the water column by stabilizing sediments from wind resuspension[35] or perhaps by direct competition with the phytoplankton[17,26]. Another factor that may have contributed to a delayed plant biomass response is localized depletion of the sedimentary seed bank due to the strong currents that occurred during the hurricane event. Research is ongoing to evaluate seed bank status in the lake. Preliminary work in-

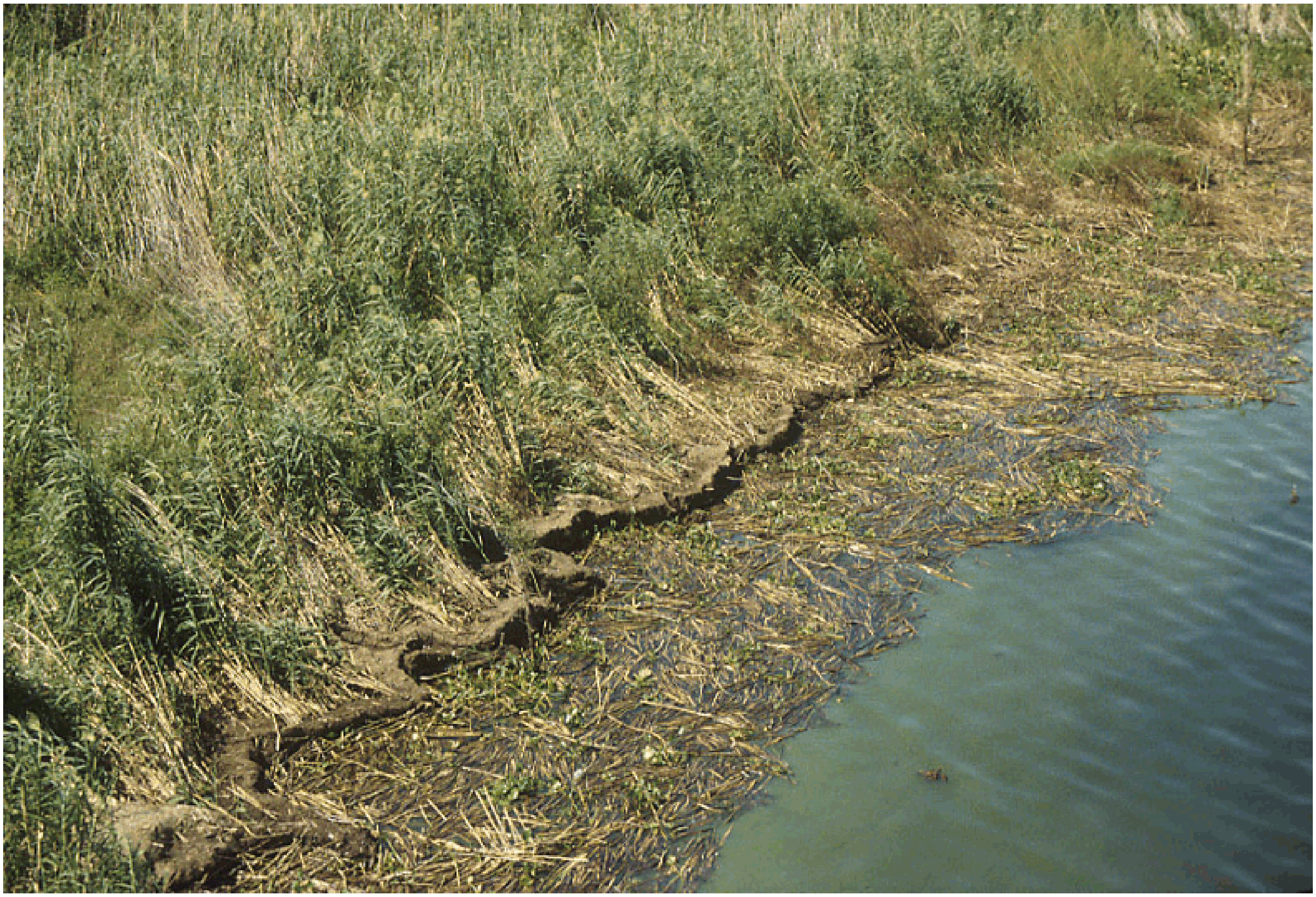

(FIGURE 4b)

dicates that some sites that supported dense *Potamogeton* beds in 1999 had little or no viable buried seeds of that plant in spring 2000. Plants may have to recolonize such areas of the lake by vegetative means, and this could be a much slower process than re-establishment from seeds.

Plant community structure also responded to the lake recession in an unexpected manner. In 1999 we found that the submerged plant community maintained a relatively constant biomass (near 230 g when summed across all sites) from April to October, with dominance by three vascular species: *Hydrilla verticillata*, *Potamogeton illinoinensis*, and *Vallisneria americana* (Fig. 5b). Following the lake recession, the biomass total increased only to 170 g in June and July. In August the biomass increased rapidly to over 600 g, but nearly 98% was due to *Chara* (Fig. 5b, 7a). Although their biomass was sparse, seedlings of vascular plants appeared in the *Chara* mat (Fig. 7b) and elsewhere around the lake starting in July. Areas where a high biomass of *Chara* occurred had extremely high water clarity, while nonvegetated areas further offshore maintained relatively turbid water. This spatial variation among vegetated and nonvegetated areas has been documented in other shallow eutrophic lakes[11].

At the end of the summer growing season there were approximately 14,000 ha of *Chara* in the near-shore zone of the lake (Fig. 8). Despite similar irradiance conditions during the middle of the growing season of 1999 and 2000, there was a distinct change from vascular plants in 2000 to *Chara* at several of the surveyed sites (Fig. 9). According to data collected during a 1988-90 survey in the lake (unpublished), *Chara* spp. are early successional plants that become dominant in formerly barren areas prior to the colonization by vascular plants. Research in the Netherlands also indicates that *Chara* spp. are pioneers in restored shallow lakes[12,36], although there are exceptions[37]. The results from Lake Okeechobee suggest that some factor occurring between 1999

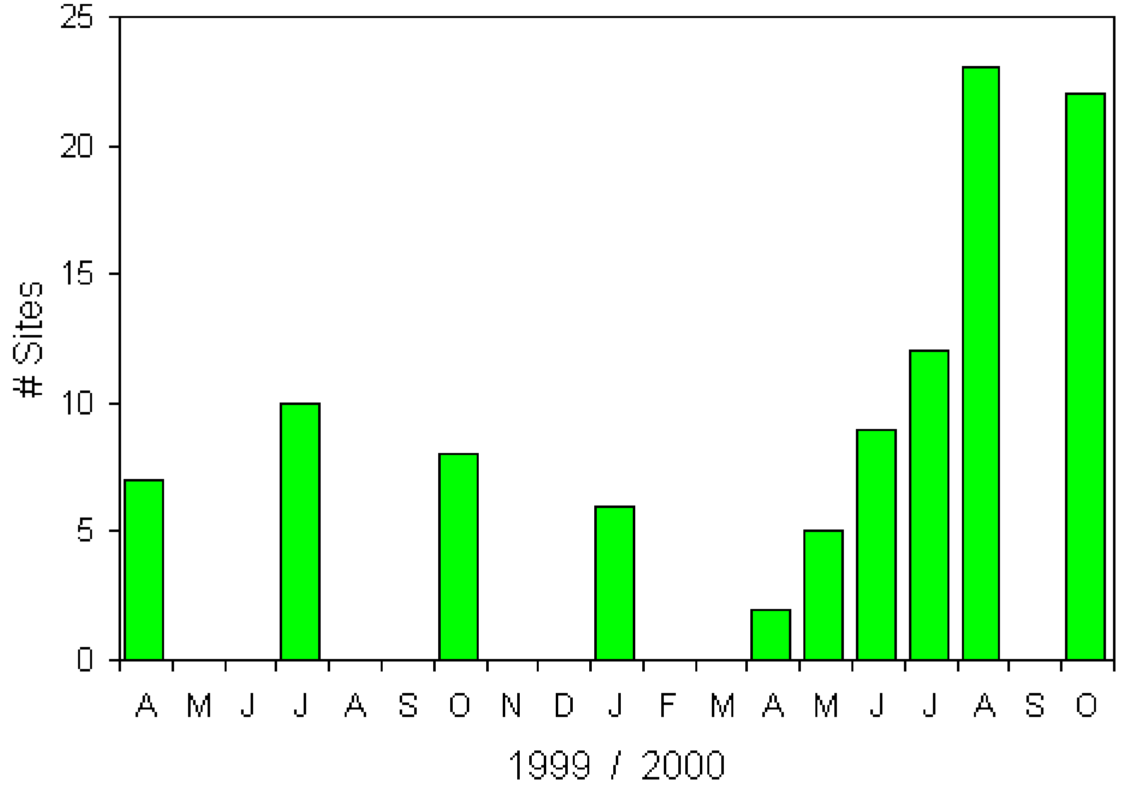

(5a)

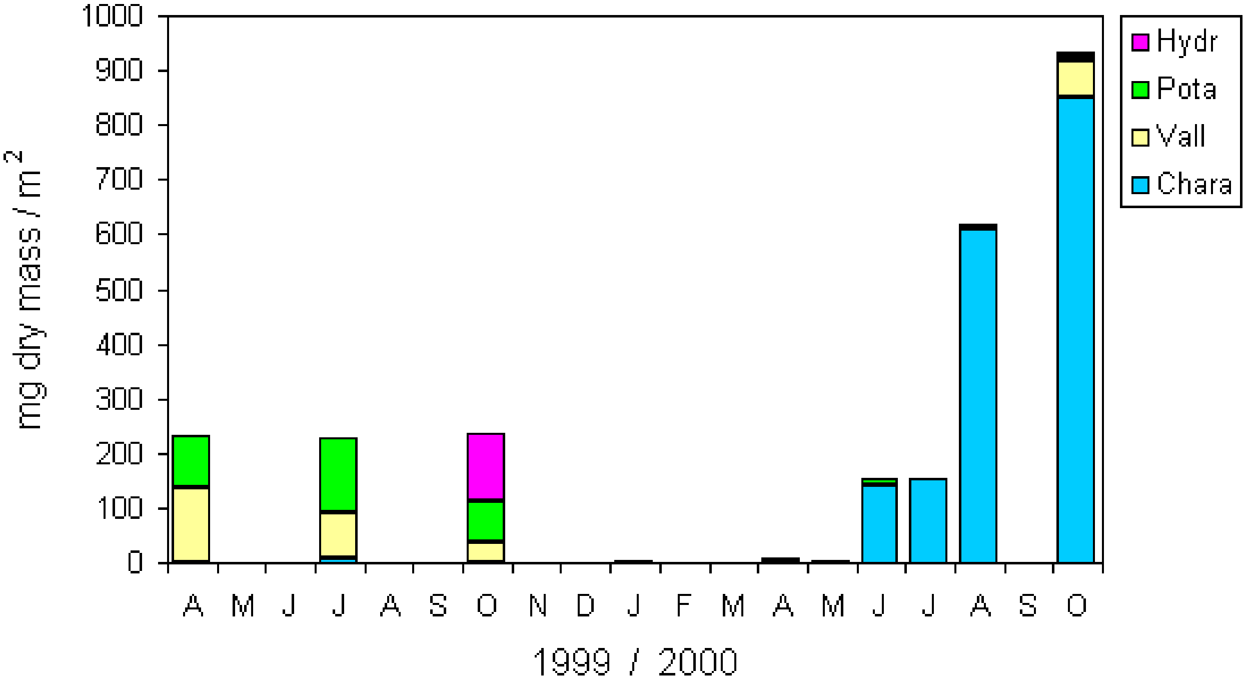

(5b)

**FIGURE 5**. Total number of sites in the 42-station submerged aquatic vegetation survey found to support plants, from April 1999 to August 2000 (a), and total biomass of various plant taxa at the sites, summed over all 42 stations, for the same time period (b).

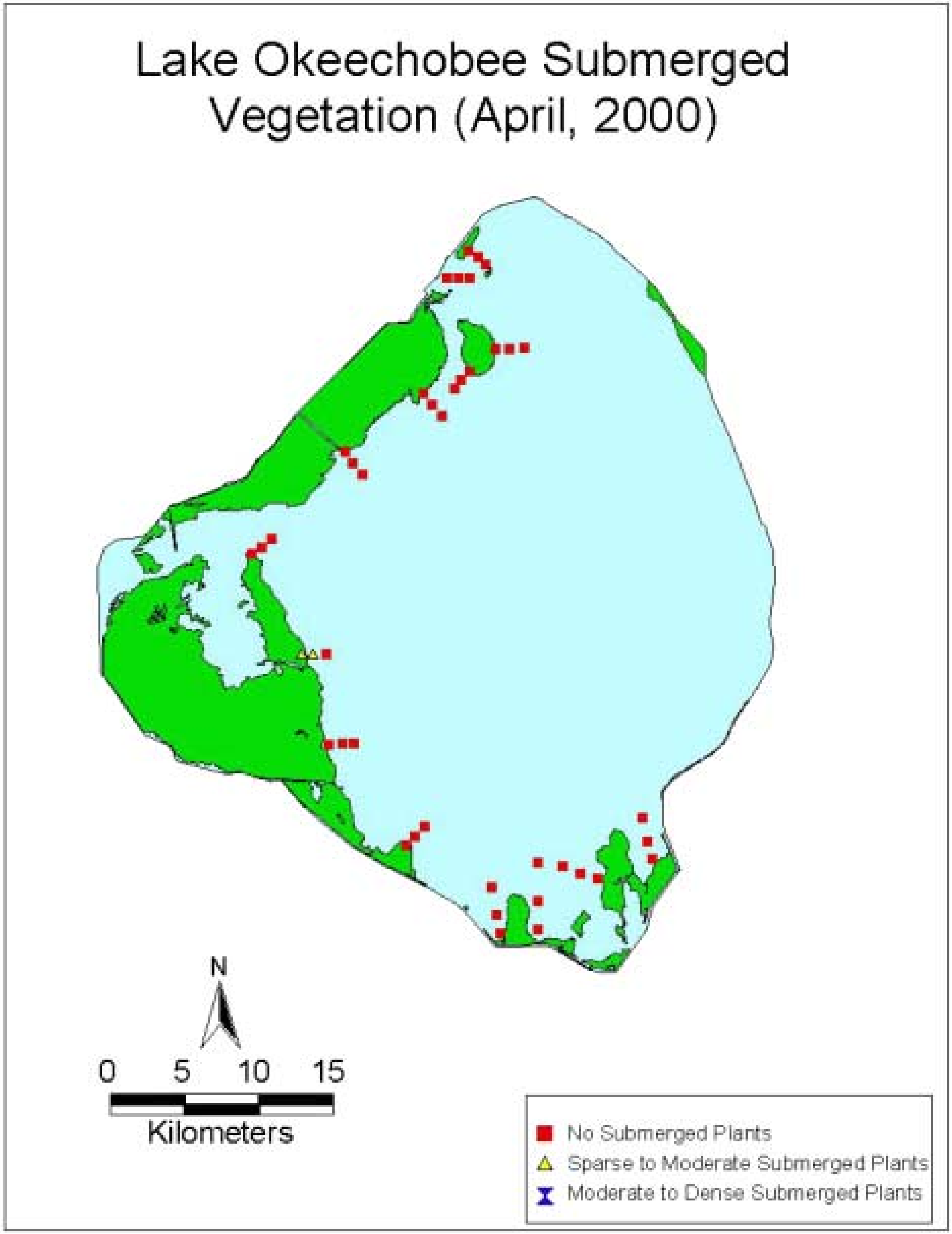

(6a)

**FIGURE 6**. Distribution of submerged aquatic vegetation in the 42-station survey during April (a) and August (b) of 2000. By comparison, only 10 sites, located primarily in the south end of the lake, contained plants at the end of summer 1999.

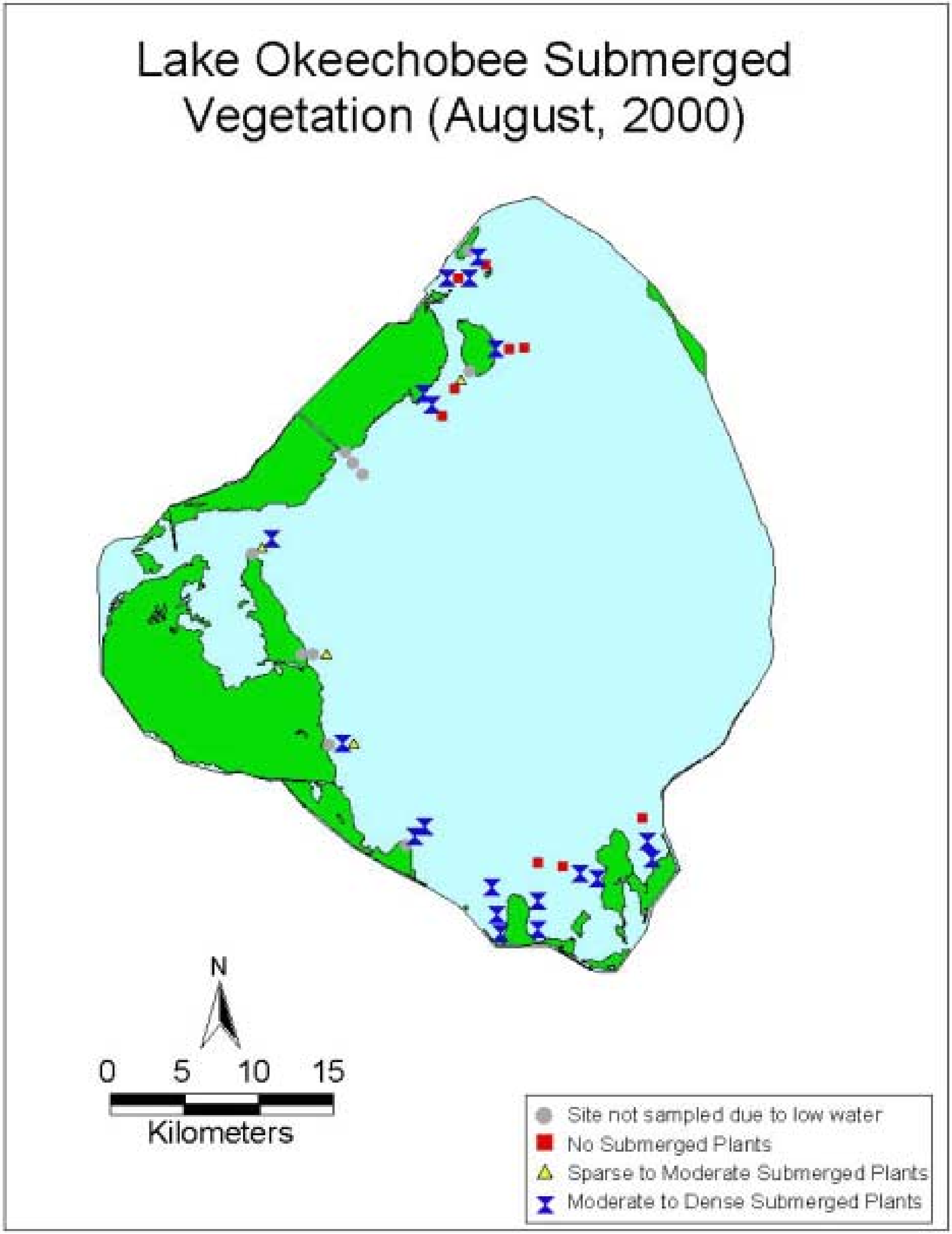

Lake Okeechobee Submerged Vegetation (August, 2000)
N
0 5 10 15
Kilometers
Site not sampled due to low water
No Submerged Plants
Sparse to Moderate Submerged Plants
Moderate to Dense Submerged Plants

**(FIGURE 6b)**

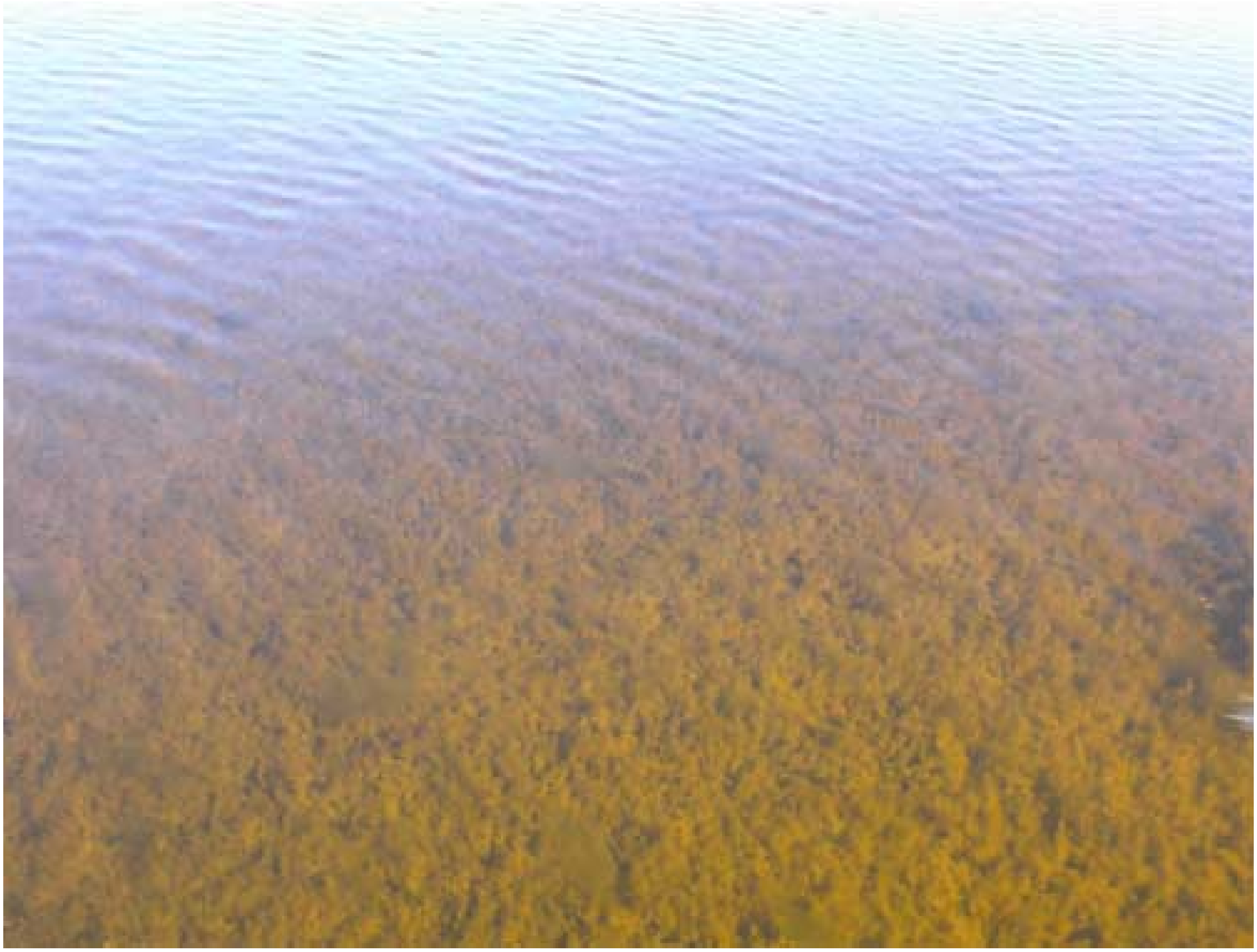

(7a)

**FIGURE 7**. Photograph of the dense lawn of *Chara* that developed over much of the near-shore region during summer 2000 (a), and a photograph of *Potamogeton* seedlings emerging from a *Chara* mat (b).

and 2000 damaged the submerged plant community, so that the recession operation gave rise to the first stages of recovery, in the form of an early successional stage.

## Effects of the Hurricane

We hypothesize that Hurricane Irene (mid-October 1999) impacted Lake Okeechobee in a manner that dramatically altered the physical, chemical, and biological conditions of the ecosystem, giving rise to the unexpected responses to the spring 2000 recession. When Hurricane Andrew impacted south Florida in 1992, its ecological impacts were studied in great detail, but were focused on marine and wetland ecosystems located 100 km south of Lake Okeechobee[38]. It has been argued that a hurricane uprooted submerged vegetation in Lake Apopka, Florida in the late 1940s, possibly switching the lake from a stable clear-water state to a stable turbid state with phytoplankton dominance[15]. However, this viewpoint is debated[39]. Ours is one of the few studies to quantify impacts of a hurricane on a shallow lake ecosystem.

In mid-October 1999, Hurricane Irene passed just to the south of Lake Okeechobee. Climatological data collected by the SFWMD at a mid-lake monitoring structure indicate that maximal wind velocities over the lake were near 90 km $h^{-1}$ and that velocities in excess of 40 km $h^{-1}$ occurred for more than 20 h (Fig. 10). We used the lake hydrodynamic / sediment transport model to simulate effects of this windstorm on current velocities and resuspended sediment concentrations during a pre- to post-storm period. This model has

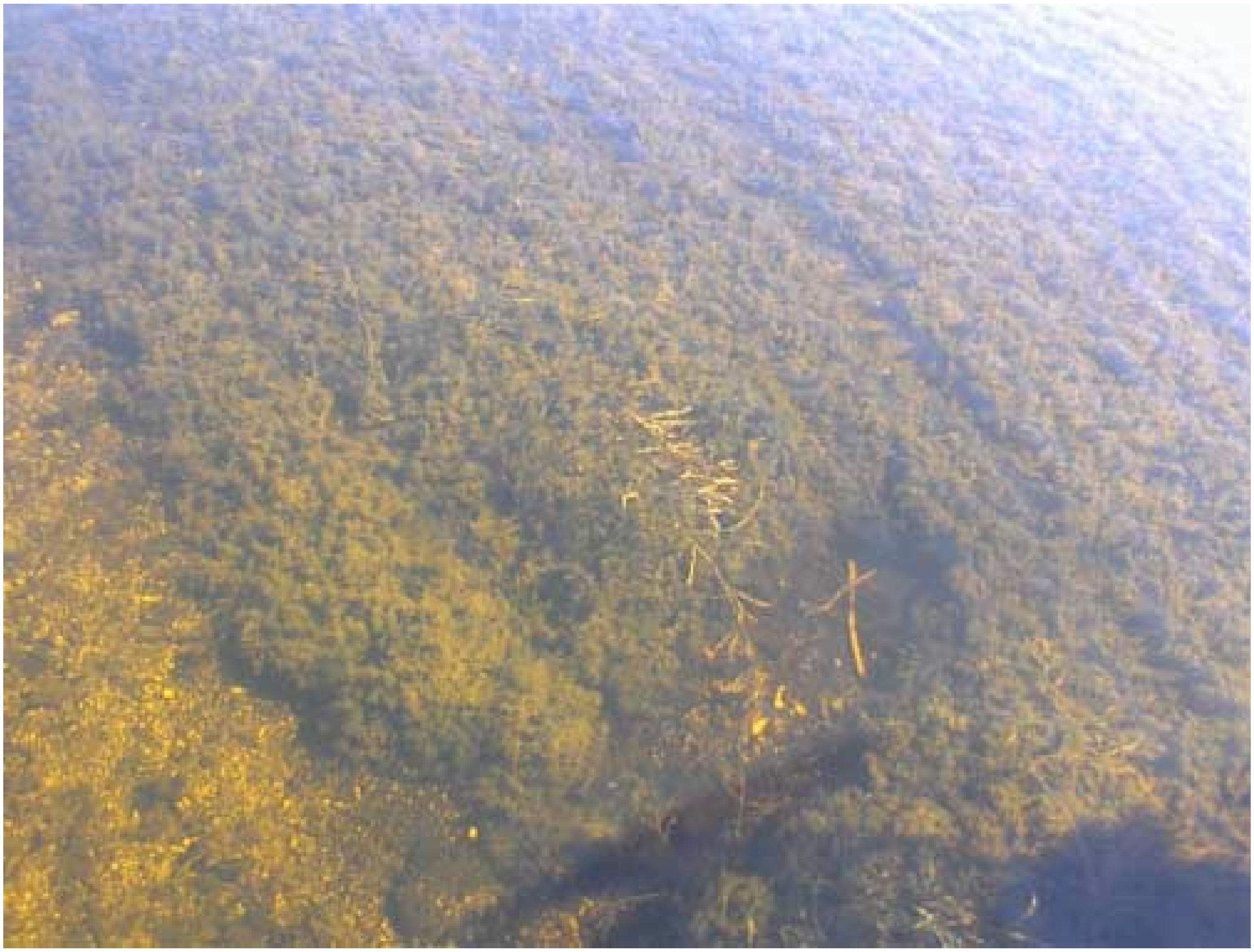

**(FIGURE 7b)**

previously been calibrated and validated (Jin et al. 2000), and it provides a good representation of conditions actually occurring in the lake under varying wind velocity and direction[21]. Output from three selected days of the model run indicates the pattern of impacts. On October 8th (Fig. 11a), prior to the storm, light winds were from the east and current velocities were below 5 cm $s^{-1}$ over much of the lake. Localized areas of greater current velocity occurred at the extreme south end of the lake and along the northwestern shoreline, where there also was a greater amount of resuspended sediment material. On October 15th (Fig. 11b), during the onset of the storm, strong winds initially were from the northeast (reflecting the counter-clockwise rotation of the hurricane). Strong surface current velocities were generated in the lake, at some locations in excess of 50 cm $s^{-1}$. At this time there was a strong clockwise circulation pattern and high amounts of resuspended sediment along the eastern shoreline and at mid-lake. On October 16th (Fig. 11c), when the storm passed to the east of the lake, strong winds were from the southwest. These winds produced one large circulation gyre in the southern half of the lake, where maximal current velocities up to 100 cm $s^{-1}$ occurred. Two smaller gyres occurred in the north and northwest. High amounts of resuspended sediment occurred along the eastern shoreline and in the extreme south. To view an animated version of the hydrodynamic model output, use this hyperlink:

http://www.sfwmd.gov/org/wrp/lakeo/animation.htm

Water quality monitoring data support the notion that Hurricane Irene had significant ecosystem-wide impacts. In early October, approximately 2 weeks prior to the storm, TP concentrations in the lake ranged from 60 to 140 µg $l^{-1}$, with a lake-wide mean of near 90 µg $l^{-1}$ (Fig. 12a). In early November, approximately 2 weeks after the storm, concentrations ranged from 120 to 240 µg $l^{-1}$ and the lake-wide mean

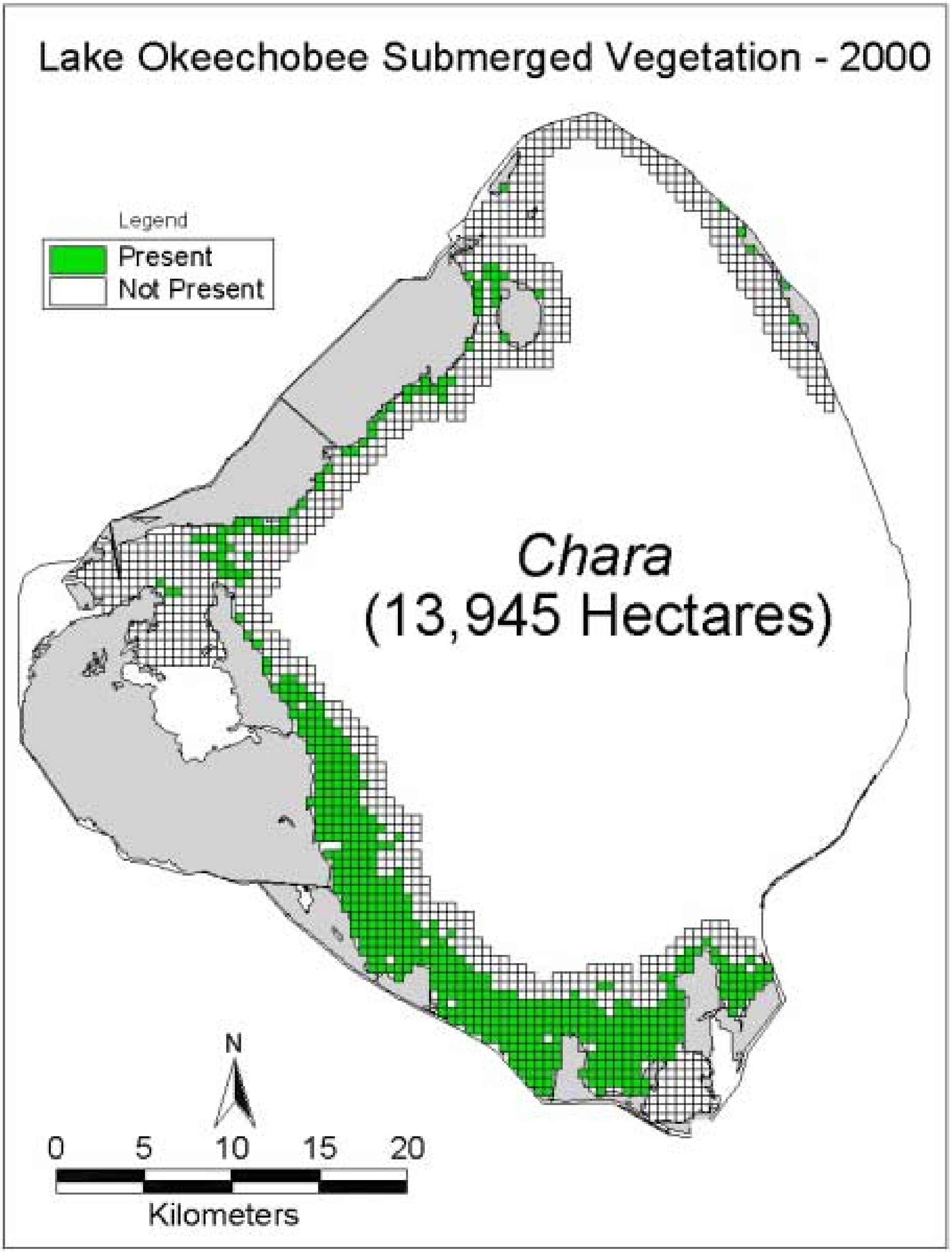

**FIGURE 8**. GIS map showing the spatial distribution of *Chara* at the end of the 2000 summer growing season in Lake Okeechobee. The survey included all of the grid cells; those found to contain *Chara* are colored green.

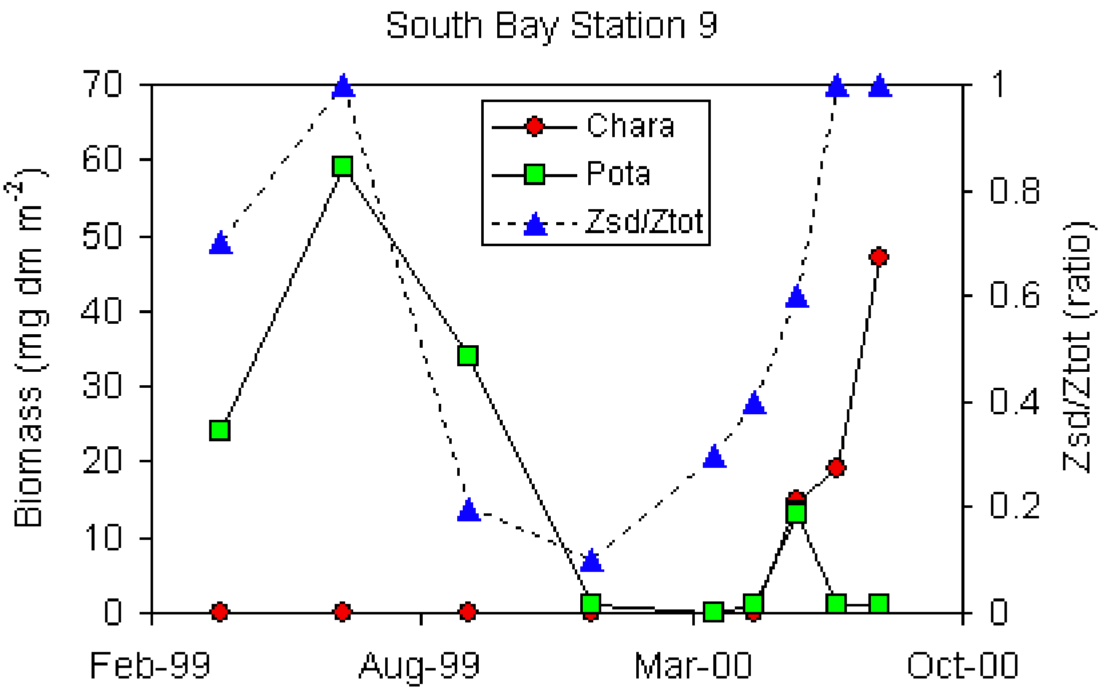

**FIGURE 9**. Typical response of the submerged plant community to events that occurred during the 1999–2000 sampling period. Sites that displayed strong dominance by vascular plants in 1999 became dominated by *Chara* in summer 2000.

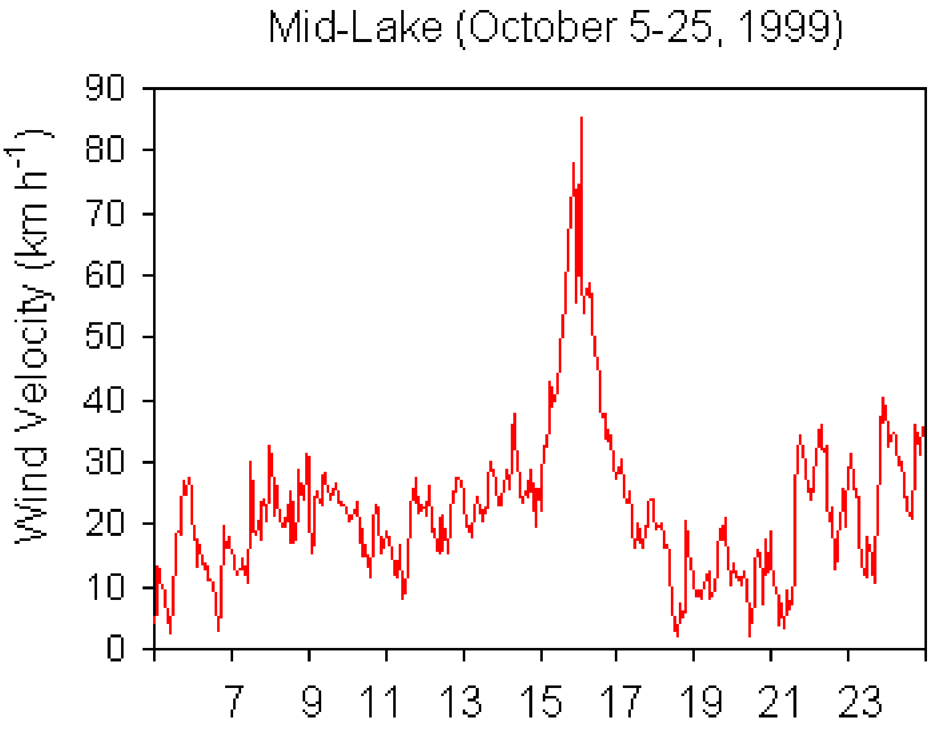

**FIGURE 10**. Wind velocities measured at a central lake meteorological station during the period from October 15 to 25, 1999, when Hurricane Irene passed across south Florida. The plotted data are hourly averages calculated from measurements taken at 15-min intervals.

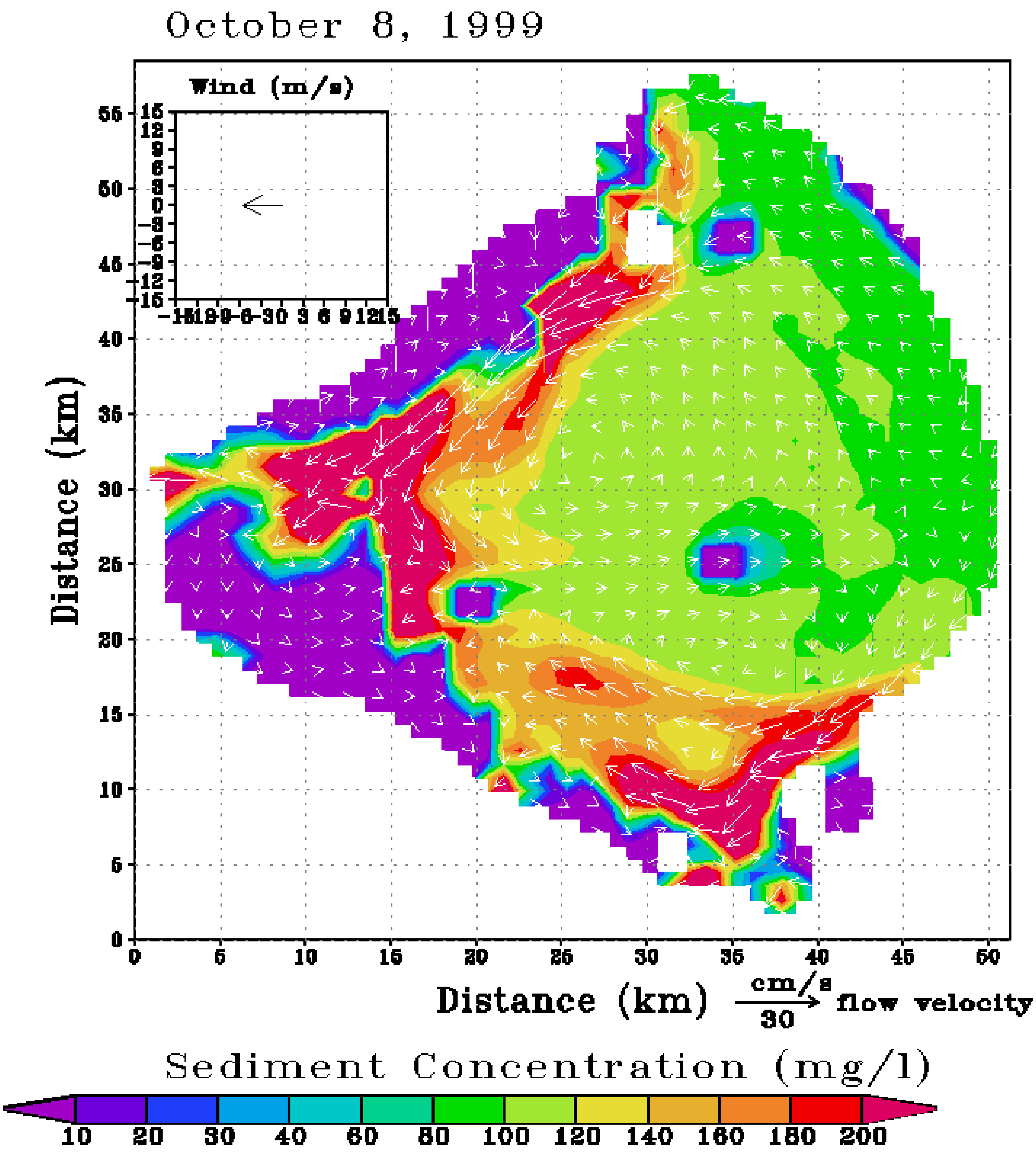

(11a)

FIGURE 11. Output from the three-dimensional lake hydrodynamic model, simulating the underwater current velocity vectors (arrows) and amounts of resuspended sediment (color scale) on a day just before Hurricane Irene impacted south Florida (a), during the initial impact of the storm (b), and during the final impact of the storm (c) as it moved away from the lake to the northeast. To view an animated version of this output go to: http://www.sfwmd.gov/org/wrp/lakeo/animation.htm.

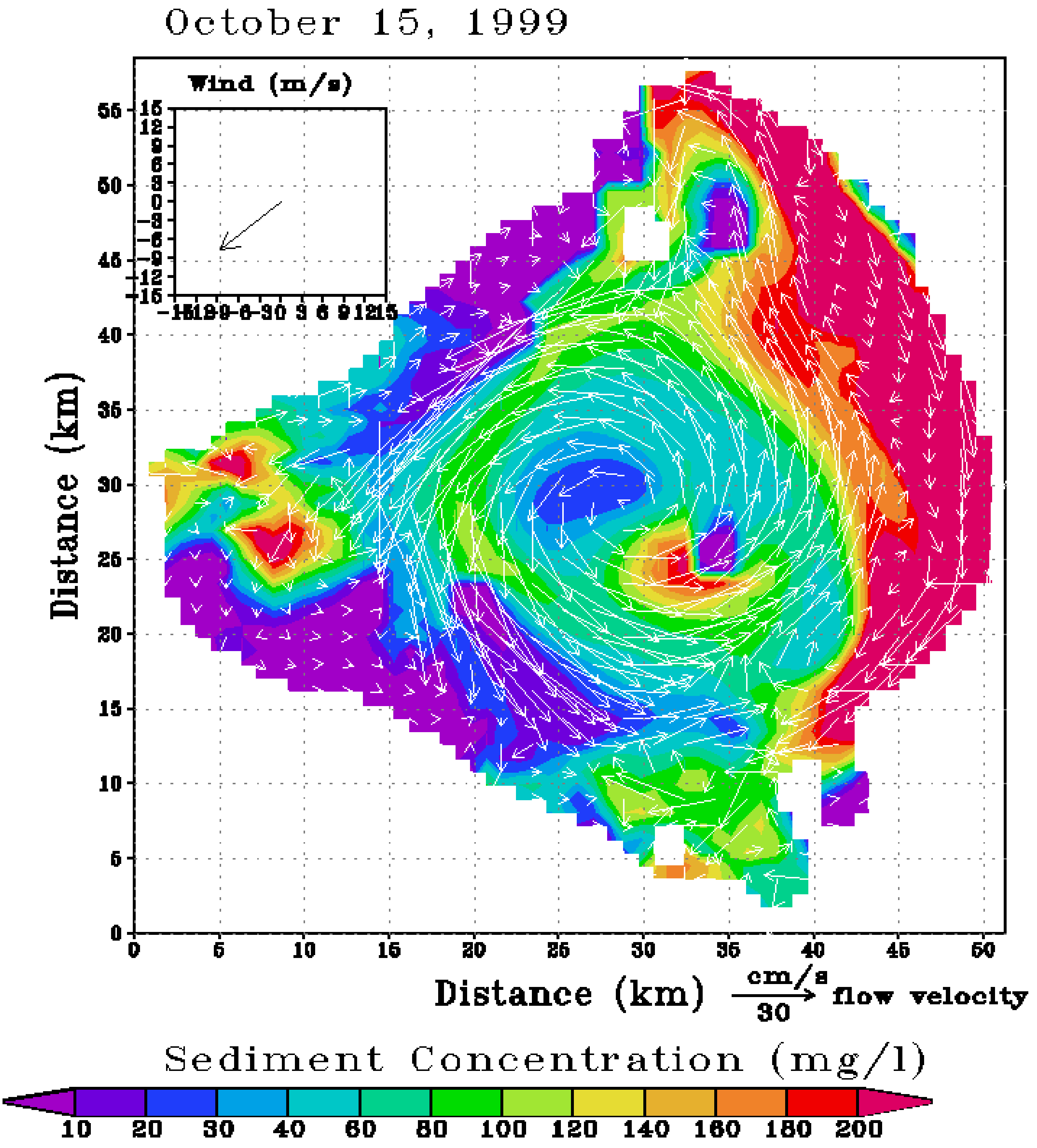

October 15, 1999
Wind (m/s)
Distance (km)
Distance (km)
cm/s
30
flow velocity
Sediment Concentration (mg/l)
10 20 30 40 60 80 100 120 140 160 180 200

(FIGURE 11b)

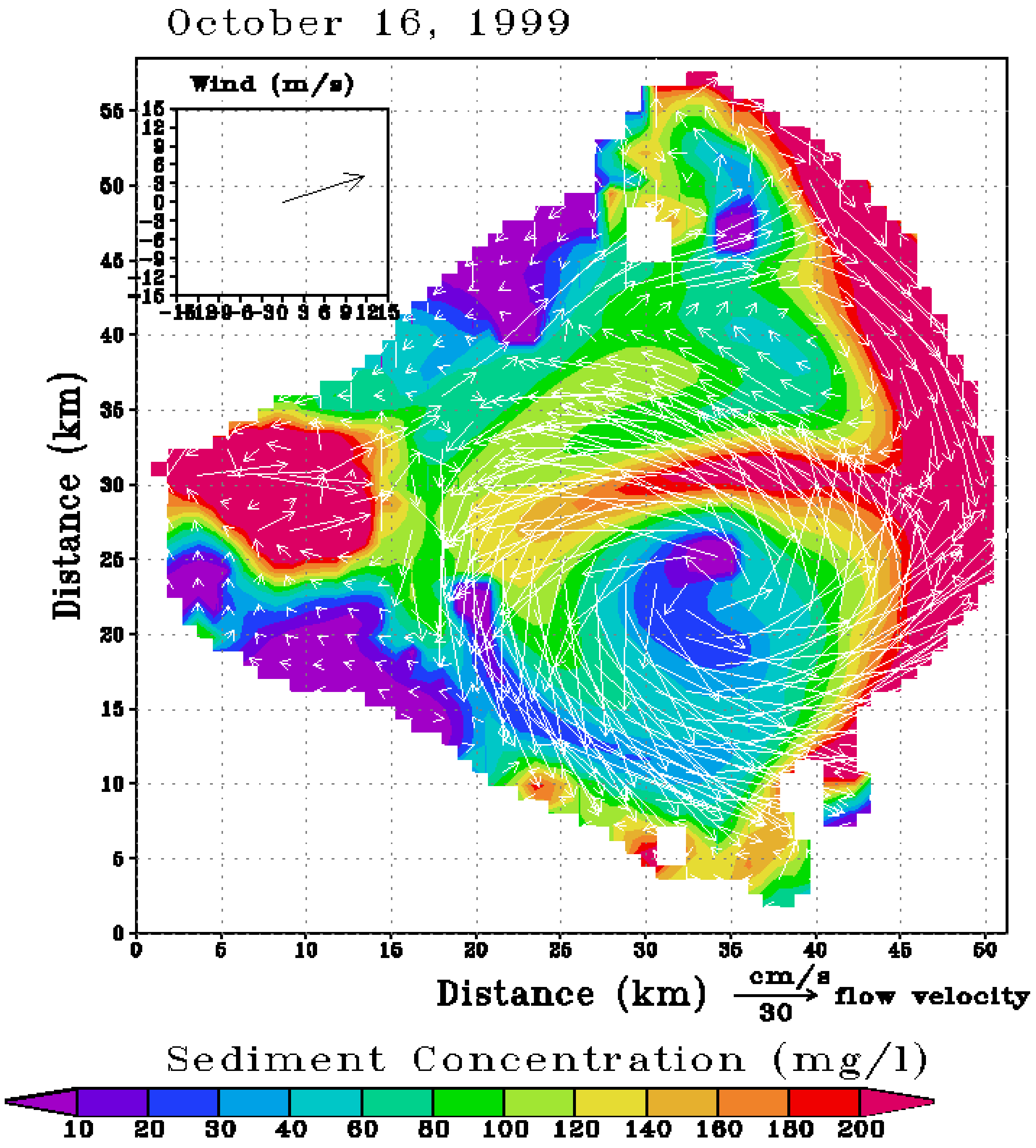

October 16, 1999
Wind (m/s)
Distance (km)
Distance (km)
cm/s
30
flow velocity
Sediment Concentration (mg/l)
10 20 30 40 60 80 100 120 140 160 180 200

(FIGURE 11c)

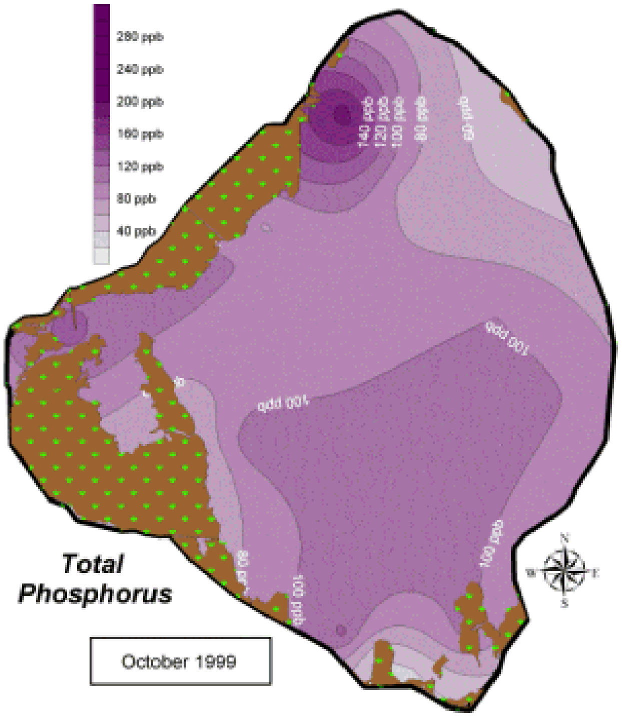

**(12a)**

**FIGURE 12**. Isoline maps showing lake-wide concentrations of total phosphorus, 2 weeks before Hurricane Irene (a) and 2 weeks afterwards (b), and corresponding maps for Secchi transparencies (c-d).

was over 220 µg $l^{-1}$ (Fig. 12b). We constructed box-and-whisker plots of pelagic mean TP concentrations based on all data collected in the "windy season" (October-March) from 1988 to 1999 and compared this with the data collected before and after the storm (Fig. 13). A similar analysis was done using data from a site located in the bay at the south end of the lake. The results indicate that the lake-wide mean prior to the storm was lower than the long-term mean, but after the storm the concentration exceeded the 95$^{th}$ percentile of past observations. The same pattern was observed at the south station, although concentrations were lower in this region. The hurricane impacts on lake-wide TP probably reflect both and resuspension of P-rich sediments from the lake bottom and increased P inputs from tributaries. Based on SFWMD inflow monitoring data, a P load of 74 metric tons was associated with high runoff that occurred in the watershed north of the lake during the passage of the storm.

Secchi depth, which is a measure of water transparency, ranged from 0.3 to 0.6 m and had a lake-wide mean of 0.4 m prior to the storm (Fig. 12c). This range and mean are typical for October, as confirmed by comparison with data from the historical period (Fig. 13). Following the storm, in early November, Secchi depth ranged from 0.1 to 0.4 m and averaged 0.2 m (Fig. 12d). This mean was below the 25$^{th}$ percentile of past observations of lake-wide means in the windy season. Data from the south station also indicate this dramatic decline in transparency, to below the 5$^{th}$ percentile of historical data. In addition to increased TP and reduced Secchi depths, there was a marked increase in TSS associated with the storm event (Fig. 13).

Turbid, nutrient-rich conditions persisted during the entire winter. A second peak in TP and TSS occurred in March, when a lesser intensity windstorm mixed the materials that likely had settled into the bottom waters but not fully consolidated into the sediments by that time. Between April and May there was a dramatic decline in TP (Fig. 14a,b) and an increase in Secchi depth (Fig. 14c,d) coincident with the lake recession operation. The improvements in lake-wide water quality were more rapid than any that had been observed in previous month-to-month comparisons of historical data, suggesting that the managed recession was the causal factor.

In regard to submerged plants, we suspect that the extremely high levels of turbidity that occurred from October to April may have considerably suppressed plant biomass. In some locations, there may have been scouring of the sedimentary seed bank and perhaps uprooting of plants by currents or burial with sediments.

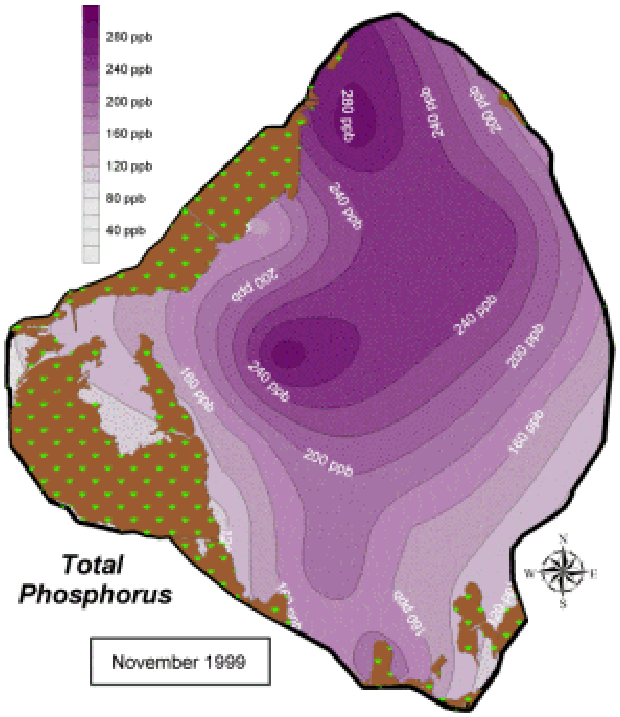

(FIGURE 12b)

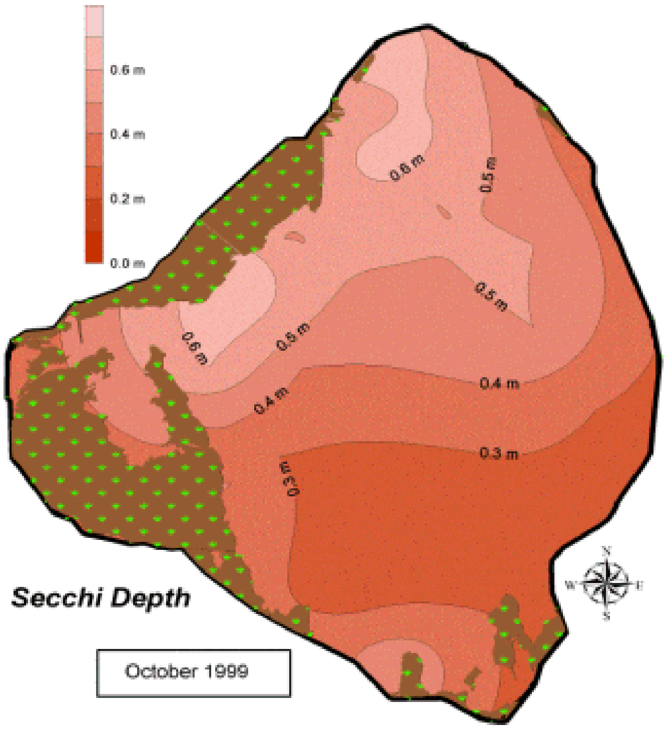

(FIGURE 12c)

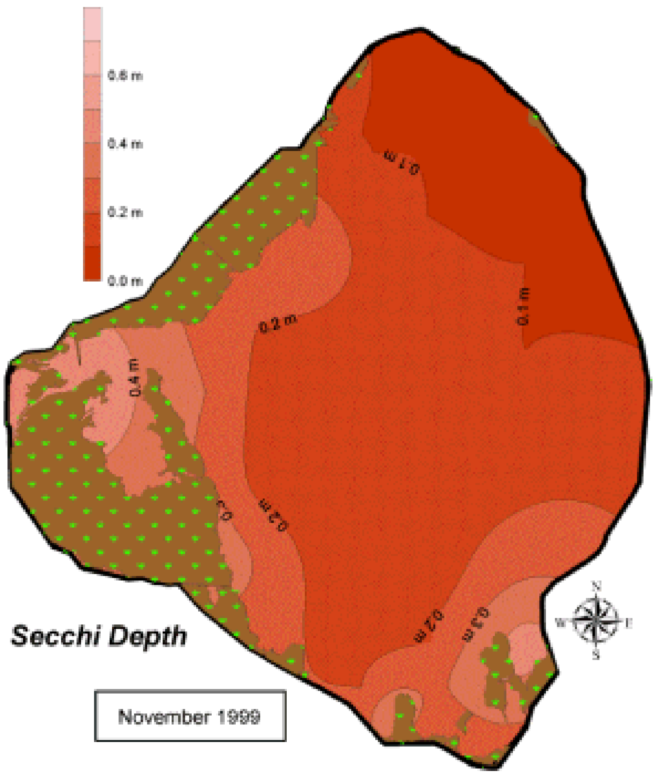

(FIGURE 12d)

## Implications for Ecosystem Management

Windstorms and other large-scale natural disturbances have the potential to affect ecosystems both in the short and long term. In the present case we documented a relatively short-term effect wherein the response of a lake to a managed recession appears to have been affected by damage caused by a storm. In this case the hurricane had relatively low wind velocities (compared to storms such as Hurricane Andrew, with velocities in excess of 300 km $h^{-1}$) and its eye did not pass directly over the lake. That the ecosystem already was heavily stressed by many years of high water and a layer of fluid mud on its bottom that could readily be resuspended by wind probably led to greater impacts than would have occurred in a less stressed system. A stronger hurricane could have even more devastating impacts, perhaps preventing the recovery that now is occurring in Lake Okeechobee.

Over a longer time frame, hurricane effects can be an important consideration for lake resource managers. In many shallow lakes around the world, management programs have aimed to improve water quality by reducing inputs of nutrients from point and nonpoint sources. Research has shown that lake recovery (in terms of reduced water column TP and phytoplankton blooms) can be delayed in shallow lakes due to buffering effects from internal nutrient loading[40], but that given enough time, shallow lakes can recover. This is expected to occur when P-rich surface sediments gradually are buried by less enriched sediments. Because internal P loading is proportional to the P concentration of active surface sediment layers, there eventually is a reduction of the internal load and improved water quality. A major hurricane could dramatically alter this natural burial, and depending on the amount of sediment material that is mixed, set back the recovery process by years or decades. If a hurricane eliminates the lake's submerged plant community for a number of years, this also could markedly impact the system's ability to assimilate incoming P loads[41]. These factors should be taken into consideration when making long-term predictions of shallow lake response to management actions in the tropics and subtropics. Clearly there is nothing that can be done to prevent hurricane impacts, but in certain circumstances they might be ameliorated. For example, sediment removal might be given greater weight as a restoration measure[42] in a system with a high probability of hurricane impact.

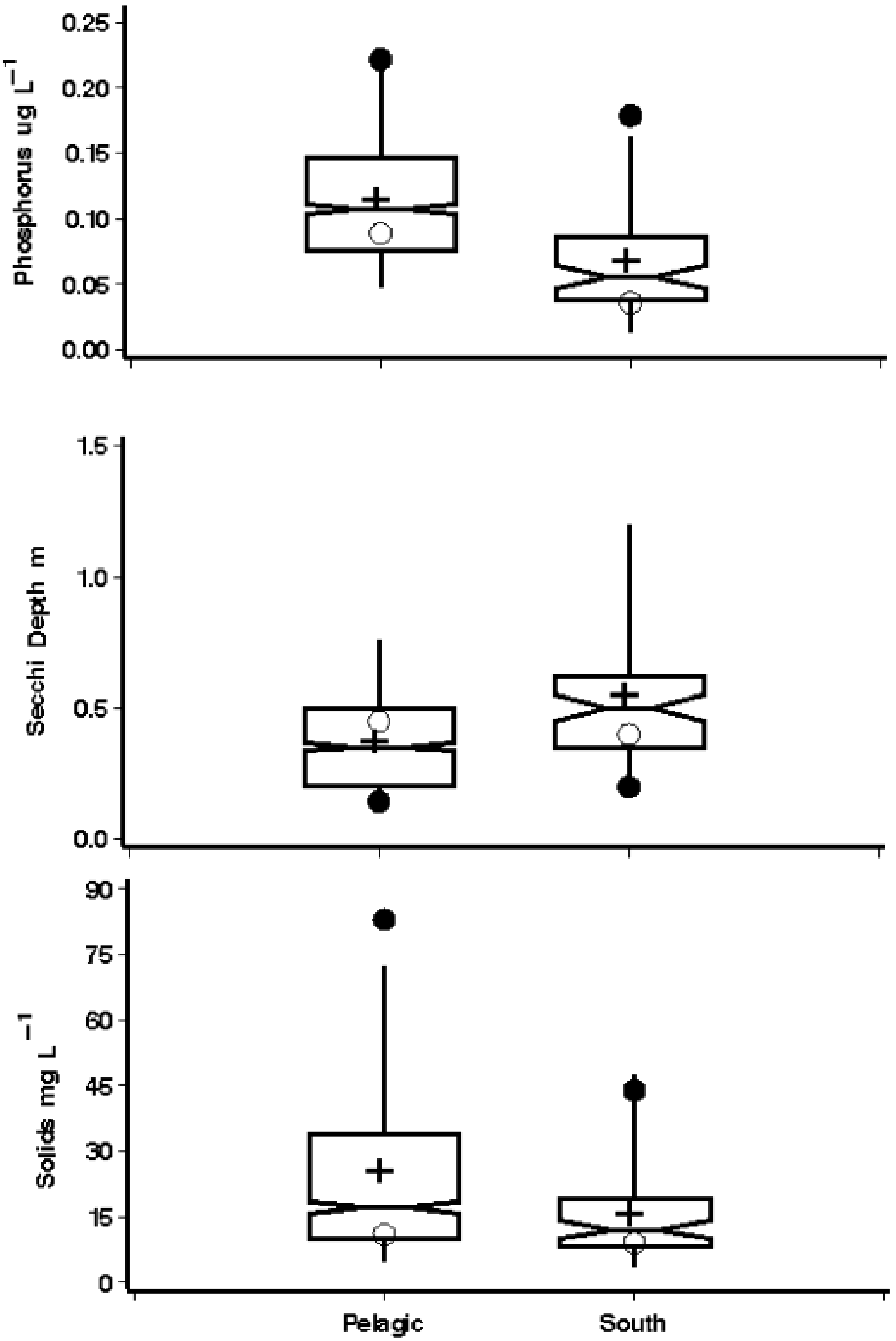

**FIGURE 13.** Box-and-whisker plots of total phosphorus concentrations, Secchi depths, and suspended solids concentrations, comparing data from 2 weeks before the storm (open circles), 2 weeks after the storm (black circles), and historical data-set from 1988 to 2000 (box-and-whiskers). Upper and lower boundaries of boxes are 75th and 25th percentiles of the historical data; lines extend to 95th and 5th percentiles; the center of each box is the median; and the + symbol indicates the historical mean. Data are shown both for the central pelagic region and the southern near-shore region of the lake.

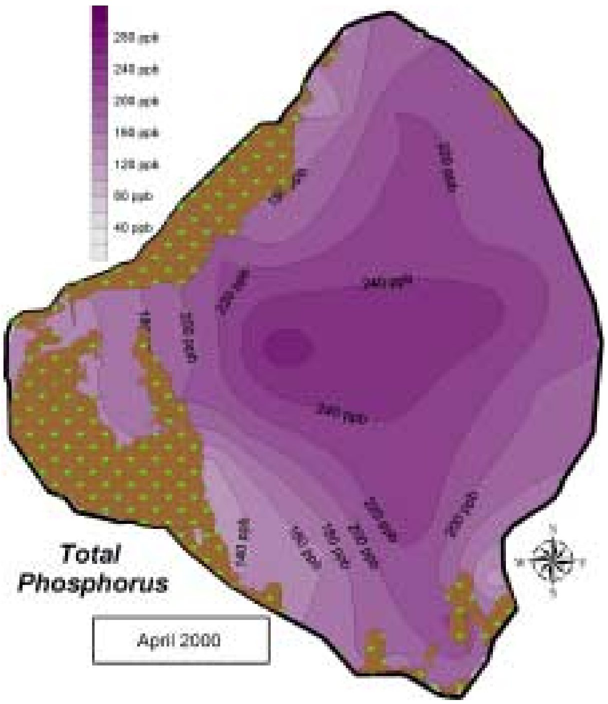

(14a)

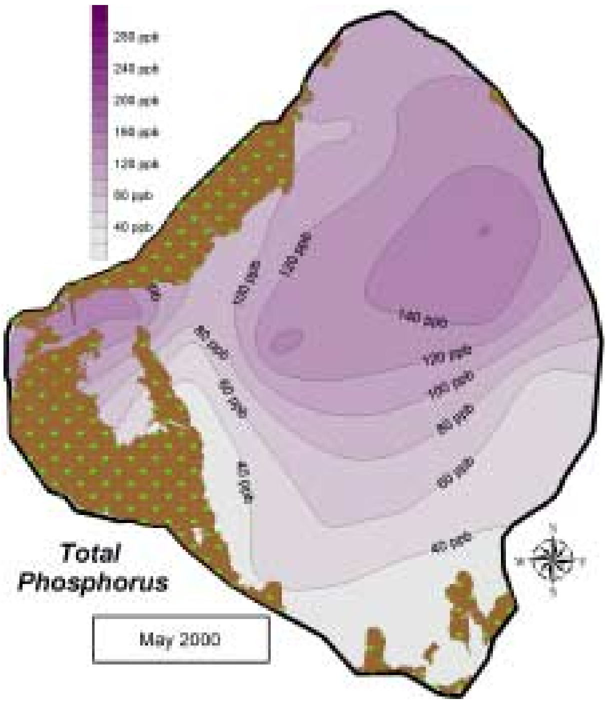

(14b)

**FIGURE 14**. Isoline maps showing lake-wide concentrations of total phosphorus, from the sampling events just prior to the spring recession operation (a) and in the month immediately afterwards (b), and corresponding maps for Secchi transparencies (c-d).

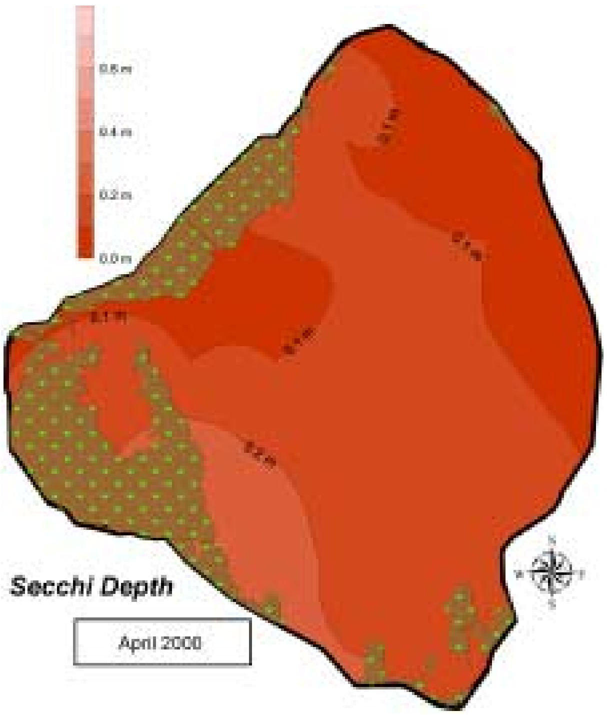

(FIGURE 14c)

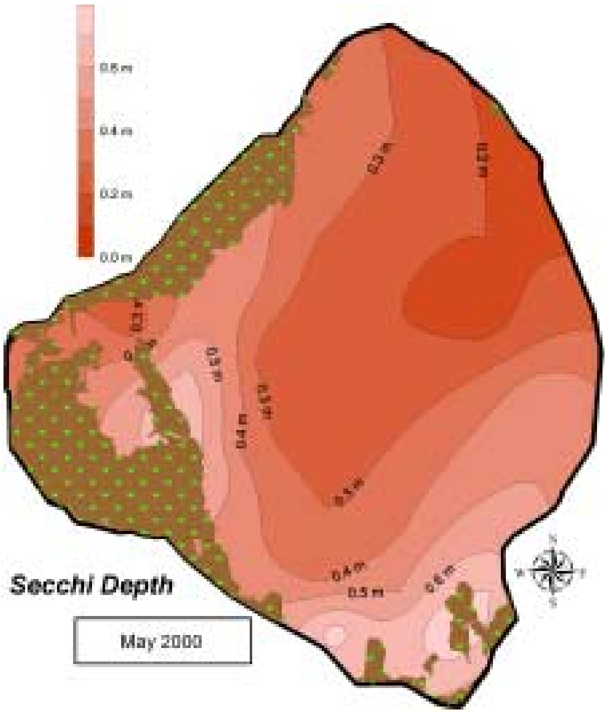

(FIGURE 14d)

## CONCLUSIONS

Scientists and managers often view ecosystems and attempt to predict their responses based on observed patterns of response to predictable external factors (e.g., seasonal variations in rainfall, evapotranspiration, and temperature). There is a growing recognition that less predictable climate patterns, such as El Niño and La Niña events, can also influence system dynamics. Additionally, in some regions of the world, stochastic events like hurricanes can exert major ecosystem impacts and affect the outcome of resource management. These events are highly unpredictable and their impacts rarely are studied. This paper offers some insight into how a hurricane could have impacted shallow Lake Okeechobee, Florida in fall 1999, by hindering the growth and perhaps directly damaging submerged plant communities, and resulting in unexpected responses to a lake recession operation aimed at improving conditions in the plant community in 2000. The hurricane essentially set back the successional process, such that in 2000 there was strong dominance by the benthic macro-alga *Chara*, in a system that previously had been dominated by vascular plants. Because water levels in the lake are expected to remain low into summer 2001, a strong recovery of the vascular plant community is likely. This is supported by the recent finding (October 2000) that there are seedlings of *Vallisneria* widely distributed around the lakeshore. Nevertheless, these results indicate the impacts that a minor hurricane can have on a shallow lake ecosystem. Stronger storms, especially if not followed by dramatic management actions such as the managed recession described here, could bring about long-term changes, perhaps even switching a shallow lake from a clear-water macrophyte-dominated condition to a turbid, phytoplankton-dominated state.

## ACKNOWLEDGMENTS

The authors are grateful to Daniel Anson, Hunter Carrick, James Grimshaw, Charles Hanlon, Ryan Maki, and Kirsten Work for providing field and laboratory assistance in this project. The paper was substantially improved as a result of constructive comments from Garth Redfield, Peter Doering, Hunter Carrick, Alan Steinman, and Susan Gray.

| | | |
|---|---|---|
| Received: | December | 22, 2000 |
| Accepted: | January | 9, 2001 |
| Published: | April | 4, 2001 |
